# Heregulin β1 drives gefitinib-resistant growth and invasion in tamoxifen-resistant MCF-7 breast cancer cells

**DOI:** 10.1186/bcr1754

**Published:** 2007-08-08

**Authors:** Iain R Hutcheson, Janice M Knowlden, Steve E Hiscox, Denise Barrow, Julia MW Gee, John F Robertson, Ian O Ellis, Robert I Nicholson

**Affiliations:** 1 Tenovus Centre for Cancer Research, Welsh School of Pharmacy, Cardiff University, Redwood Building, King Edward VII Avenue, Cardiff CF10 3XF, UK; 2 Professorial Unit of Surgery, Nottingham City Hospital, Hucknall Road, Nottingham, NG5 1PB, UK

## Abstract

**Introduction:**

Resistance to anti-epidermal growth factor receptor (anti-EGFR) therapies is an emerging clinical problem. The efficacy of anti-EGFR therapies can be influenced by the presence of heregulins (HRGs), which can bind erbB3/4 receptors and can activate alternative signalling pathways. In the present study we have examined whether HRG signalling can circumvent EGFR blockade in an EGFR-positive tamoxifen-resistant MCF-7 (Tam-R) breast cancer cell line.

**Methods:**

Tam-R cells, incubated with the selective EGFR tyrosine kinase inhibitor gefitinib ('Iressa', ZD1839), were exposed to HRGβ1 and the effects on erbB receptor dimerization profiles and on activation of associated downstream signalling components were assessed by immunoprecipitation, western blotting and immunocytochemistry. The effects of HRGβ1 on gefitinib-treated Tam-R cell growth and invasion were also examined, and HRGβ1 expression levels were assessed in breast cancer tissue by immunohistochemistry to address the potential clinical relevance of such a resistance mechanism.

**Results:**

In Tam-R cells, HRGβ1 promoted erbB3/erbB2 and erbB3/EGFR heterodimerization, promoted ERK1/2 and AKT pathway activation and increased cell proliferation and invasion. Gefitinib prevented HRGβ1-driven erbB3/EGFR heterodimerization, ERK1/2 activation and Tam-R cell proliferation, but HRGβ1-driven erbB3/erbB2 heterodimerization, AKT activation and Tam-R cell invasion were maintained. A combination of gefitinib and the phosphatidylinositol 3-kinase inhibitor LY294002 effectively blocked HRGβ1-mediated intracellular signalling activity, growth and invasion in Tam-R cells. Similarly, targeting erbB2 with trastuzumab in combination with gefitinib in Tam-R cells reduced HRGβ1-induced erbB2 and ERK1/2 activity; however, HRGβ1-driven AKT activity and cell growth were maintained while cell invasion was significantly enhanced with this combination. In clinical tissue all samples demonstrated cytoplasmic tumour epithelial HRGβ1 protein staining, with expression correlating with EGFR positivity and activation of both AKT and ERK1/2.

**Conclusion:**

HRGβ1 can overcome the inhibitory effects of gefitinib on cell growth and invasion in Tam-R cells through promotion of erbB3/erbB2 heterodimerization and activation of the phosphatidylinositol 3-kinase/AKT signalling pathway. This may have implications for the effectiveness of anti-EGFR therapies in breast cancer as HRGβ1 is enriched in many EGFR-positive breast tumours.

## Introduction

The epidermal growth factor receptor (EGFR), a member of the erbB proto-oncogene family of receptor tyrosine kinases, which also includes erbB2, erbB3 and erbB4, is a transmembrane glycoprotein composed of an extracellular ligand-binding domain and an intracellular domain containing tyrosine kinase activity [1,2]. Activation of EGFR results from binding of epidermal growth factor-related growth factors, such as epidermal growth factor, transforming growth factor alpha (TGFα) and amphiregulin, which induce receptor homodimerization and/or heterodimerization with other members of the erbB receptor family [2]. No direct ligand for erbB2 has yet been identified; however, erbB2 plays a central role in erbB receptor function as it is the preferred dimerization partner for all other erbB family members [3,4].

Receptor dimerization stimulates the intrinsic receptor tyrosine kinase activity and promotes autophosphorylation of tyrosine residues within the cytoplasmic domain of the receptor. These phosphotyrosine residues provide docking sites for a variety of adaptor proteins and enzymes involved in the recruitment and activation of downstream intracellular signalling cascades, including the mitogen-activated protein kinase (MAPK) and phosphatidylinositol-3 kinase (PI3K) pathways [2]. These signalling cascades can promote proliferation, angiogenesis and invasion, and can inhibit apoptosis, key mechanisms underlying tumour growth and progression [5]. This oncogenic potential in conjunction with the aberrant expression and/or activation of EGFR, which has been reported in a range of human malignancies including breast cancer, provides a strong rationale for targeting this growth factor receptor [6,7].

A number of agents targeting EGFR have now been developed and include the monoclonal antibody cetuximab, which targets the extracellular ligand-binding domain of EGFR, and the small molecule tyrosine kinase inhibitors gefitinib (Iressa, ZD1839) and erlotinib (Tarceva, OSI-774), which competitively block binding of ATP to the tyrosine kinase domain of the receptor [8]. These compounds are proven effective antitumour agents as monotherapies in both the preclinical and clinical setting, and have been shown to enhance the effects of cytotoxic agents and radiation when utilized in combination with these conventional chemotherapies [8-10]. Consequently, cetuximab, gefitinib and erlotinib have now all gained approval for cancer treatment in the clinic. Recent findings from clinical trials, however, have revealed that only a small cohort of patients have derived significant benefit from these therapies, with both *de novo *and acquired resistance to these agents being evident [11-13]. Furthermore, evidence of resistance to anti-EGFR therapies has now also been reported in preclinical cell models [12,13]. A number of potential resistance mechanisms have now been implicated, including receptor mutation, loss of downstream effector components and activation of alternative oncogenic signalling pathways [12,14].

A common resistance mechanism to anti-EGFR therapies identified by a number of research groups in preclinical cancer models of the colon, the breast, the prostate and the brain involves activation of the PI3K/AKT signalling pathway either as a consequence of the loss of phosphatase and tensin homologue or of increased insulin-like growth factor type 1 receptor activity [12,15-19]. PI3K/AKT signalling pathway activity can also be driven through activation of erbB3 and erbB4 receptors via their ability to directly recruit the p85 regulatory subunit of PI3K [20,21]. erbB3 and erbB4 are receptors for the neuregulin family of growth factors, which consists of NRG1 or heregulin (HRG), NRG2, NRG3 and NRG4. These neuregulins have been shown to potently drive cell proliferation, survival, motility and invasion in a range of cancer cell types [22]. In breast cancer, HRG has been reported to be detectable in clinical tissue [23,24] and has been identified as a mitogenic and proinvasive factor in preclinical studies both *in vitro *and *in vivo *[25-31].

Previous studies from our group have established that growth of a tamoxifen-resistant MCF-7 (Tam-R) breast cancer cell line is driven by the autocrine release of the epidermal growth factor-like ligand amphiregulin and activation of the EGFR/MAPK signalling pathway [32-34]. Furthermore, activation of this signal transduction mechanism has also been implicated in the increased motility and invasiveness observed in this cell line [35]. Both Tam-R cell growth and invasion are therefore sensitive to the inhibitory actions of the selective EGFR tyrosine kinase inhibitor gefitinib [32-35]. In the present study we have investigated whether HRGβ1 can overcome the inhibitory effects of gefitinib on Tam-R cell growth and invasion, and we have examined the potential pathways activated by this erbB3/4 ligand. We have further assessed the expression levels of HRGβ1 in a series of 77 primary breast cancer samples from patients who had received no prior therapy to assess whether such signalling may be important in the clinical setting.

## Materials and methods

### Cell culture

All tissue culture medium and constituents were purchased from Gibco Europe Ltd. (Paisley, UK), and tissue culture plastics were obtained from Nunc (Roskilde, Denmark). The EGFR-positive tamoxifen-resistant Tam-R cell line was developed by continually exposing MCF-7 breast cancer cells, a gift from AstraZeneca Pharmaceuticals (Cheshire UK), to 4-hydroxytamoxifen (100 nM) over a period of 6 months [32]. The Tam-R cell line was maintained in phenol-red-free RPMI medium containing 5% charcoal-stripped steroid-depleted FCS, penicillin–streptomycin (10 IU/ml–10 μg/ml), fungizone (2.5 μg/ml), glutamine (4 mM) and 4-hydroxytamoxifen (100 nM in ethanol). This cell line was maintained at 37°C in a humidified 5% CO_2 _atmosphere.

### Immunoprecipitation and western blotting

#### Experimental cell culture

The Tam-R cell line was grown for 4 days before being transferred into phenol-red/steroid-free, serum growth factor-free DCCM (Biosynergy (Europe), Cambridge, UK) for 24 hours. The cells were then lysed for protein or mRNA analysis. To examine the effects of pharmacological agents, cells were lysed following a further incubation in DCCM supplemented with either HRGβ1 (10 ng/ml in ethanol; Sigma, Poole, UK) for 10 minutes, gefitinib (1 μM in ethanol; AstraZeneca, Macclesfield, UK) for 1 hour, trastuzumab (100 nM in water; Roche Pharmaceuticals, Penzberg, Germany) for 7 days or LY 294002 (10 μM in water; Affiniti Research Products Ltd, Exeter, UK) for 1 hour, or combinations of these treatments. Controls were incubated for the same periods of time with the appropriate vehicle. All experiments were performed at least three times.

#### Cell lysis

Cells were washed three times with PBS and lysed using ice-cold lysis buffer (for composition, see Knowlden and colleagues [32]. The cellular contents were transferred to microfuge tubes, clarified by centrifugation at 13,000 rpm for 15 minutes at 4°C and the supernatant aliquots stored at -20°C until required. Total protein concentrations were determined using the DC BioRad protein assay kit (BioRad Labs Ltd, Hemel Hempstead, UK).

#### Immunoprecipitation

Cell lysates containing 1 mg protein were immunoprecipitated using 1 μg specific total EGFR and erbB3 antibody and were incubated on ice for 1 hour. Then 30 μl protein A agarose (Insight Biotechnology Ltd, Wembley, UK) was added to the mixture and the mixture was inverted frequently by hand for a further 2 hours. The immune complex was centrifuged at 3,000 rpm at 4°C for 5 minutes and was washed with ice-cold lysis buffer. This procedure was repeated twice and the resultant pellet was resuspended in 20 μl Laemmli sample loading buffer containing 0.01 M dithiothreitol. Samples were heated to 100°C for 5 minutes to release the bound proteins prior to gel loading.

#### Western blotting

Protein samples from total cell lysates (20 μg) were subjected to electrophoresis separation on a 7.5% polyacrylamide gel and were transblotted onto a nitrocellulose membrane (Schleicher and Schuell, Dassel, Germany). Blots were blocked at room temperature for 1 hour in 5% skimmed milk powder made up in Tris-buffered saline (TBS)–Tween 20 (0.05%), and were incubated for a minimum of 1 hour in primary antibody diluted 1/40,000 for β-actin (reference control) or 1/1,000 for EGFR, erbB2, erbB3, erbB4, AKT and ERK1/2 MAPK in 1% marvel/TBS–Tween. The membranes were washed three times in TBS–Tween and then incubated for 1 hour with the required secondary IgG horseradish peroxidase-labelled donkey anti-rabbit or sheep anti-mouse (Amersham Biosciences UK Ltd, Buckinghamshire, UK), diluted 1/20,000 in 1% marvel/TBS–Tween. Detection was performed using West Dura chemiluminescent detection reagents (Pierce and Warriner Ltd, Chester, UK).

The antibodies used were total EGFR (SC-03) erbB2 (SC-284), erbB3 (SC-285) and erbB4 (SC-283) (Insight Biotechnology Ltd), anti-phospho-erbB2 (pY1248, 2247), anti-phospho-EGFR (pY1068, 2234), total AKT (9272), phospho-AKT (pS473, 9271), total ERK1/2 (9102) and phospho-ERK1/2 (pT202/pY204, 9101) (New England Biolabs, Hitchin, Hertfordshire, UK), and β-actin (AC-15) (Sigma). These antibodies were selected as they have been demonstrated to be monospecific and do not cross-react with other family members. Results were scanned using a BioRad model GS-690 Imaging Densitometer (BioRad Labs Ltd, Hemel Hempstead, Hertfordshire, UK).

#### Reverse transcriptase-polymerase chain reaction

Total RNA was isolated from Tam-R breast cells and DU145 prostate cancer cells grown under basal conditions, using an RNA isolator kit (Genosys Biotech Inc., Cambridge, UK), and 1 μg was reverse transcribed using standard conditions as described previously [32]. Sterile water was also used, in place of RNA, as a negative control for reverse transcription. Resultant cDNA samples, reverse transcription negative control samples and sterile water (negative PCR control) samples were amplified for 40 cycles using specific primers for HRGβ1 and the conditions were optimized as described previously [32].

The primers used for HRGβ1 were designed manually, and the specificity was checked with the EMBL-GenBank database software using the Blast program. The HRGβ1 primers were 5'-GAT CAT CAC TGG TAT GCC AG and 3'-TAA ATT CAA TCC CAA GAT GC.

### Cell invasion assay

Cell invasion was quantified using a modification of a previously described method [36]. Briefly, ThinCert tissue cell culture inserts (31.2 mm^2 ^culture surface, 8.0 μm pore size; Greiner Bio-One, Gloucester, UK) were coated with Matrigel (0.4 μg/ml) overnight at room temperature in a sterile tissue culture hood. After rehydrating the matrix with serum-free RPMI for 1 hour at 37°C, cells were seeded into the chambers at 5 × 10^4 ^cells/well, while 500 μl culture medium were added to the outside of the well. Cells were incubated with either HRGβ1 (10 ng/ml), gefitinib (1 μM), trastuzumab (100 nM), LY 294002 (10 μM) or combinations of these treatments. Controls were incubated for the same periods of time with the appropriate vehicle. Inserts were cultured at 37°C for 72 hours, after which the noninvasive cells and Matrigel were removed from the inside of the wells with a cotton swab. After fixation of the invaded cells with 4% formaldehyde, porous membranes were removed from the invasion chamber using a scalpel blade and were mounted onto glass microscope slides using Vectashield (Molecular Probes, Eugene, OR, USA) containing the nuclear stain 4'-6-diamidino-2-phenylindole. Cell invasion was quantified with a fluorescent microscope by viewing five separate fields per membrane at 20-times magnification and counting the number of cells (identified by stained cell nuclei) in each field. Data were then plotted as mean cells per field ± standard deviation.

### Cell proliferation

Cell monolayers were grown for 7 days in serum growth factor-free DCCM in the presence of either HRGβ1 (10 ng/ml), gefitinib (1 μM), trastuzumab, (100 nM), LY 294002 (10 μM), combinations of these agents or the appropriate vehicle control. Cell population growth was then evaluated by means of trypsin dispersion of the cell monolayers and the cell number was measured using a Coulter counter (Beckman, Luton, UK). All experiments were performed in triplicate.

### Clinical tissue and immunocytochemistry

#### Clinical samples

A series of 77 primary tumours were excised from patients with histologically proven breast cancer presenting for surgery at the City Hospital, Nottingham during the period 1984–1987. Representative tissue samples from these 77 samples were fixed routinely in 4% formal saline and embedded in paraffin. No patient had previously received any form of adjuvant endocrine or cytotoxic therapy. Parallel data for the histologic grade of malignancy, the menopausal status, the site of disease and the Ki-67 index, together with information on nuclear oestrogen receptor, membrane EGFR, membrane erbB2, membrane erbB3, cytoplasmic TGFα and nuclear phosphorylated MAPK protein, were available for these samples (Table 1). This research was approved by Nottingham Research Ethics Committee 2 under the title 'Development of a molecular genetic classification of breast cancer' (C2020313).

**Table 1:** Clinicopathological parameters for the clinical tumour set

	Number	Percentage
Tumour grade		
Grade 1	2	2.6
Grade 2	29	37.7
Grade 3	43	55.8
Not known	3	3.9
Oestrogen receptor status		
Positive	46	59.7
Negative	31	40.3
Epidermal growth factor receptor (membrane) status		
Positive	43	55.8
Negative	31	40.3
Not known	3	3.9
erbB2 (membrane) status		
Positive	19	71.4
Negative	55	24.7
Not known	3	3.9
erbB3 (membrane) status		
Positive	61	79.3
Negative	9	11.7
Not known	7	9.1
Menopausal status		
Premenopausal	23	31.1
Postmenopausal	51	68.9
Not known	3	3.9
Site of disease		
Locally advanced	29	39.2
Metastatic	45	60.8
Not known	3	3.9
Ki-67 index		
0	10	13
<10	15	19.5
10–30	52	67.5

#### Immunocytochemistry

Paraffin wax sections from each tumour sample from the series of 77 patients were dewaxed using xylene treatment and were then rehydrated through graded ethanols to PBS. Endogenous peroxidases were destroyed by immersing the sections in 3%hydrogen peroxide prepared in methanol, followed by rinsing with distilled water. Prior to blocking with either BSA or goat serum, antigen retrieval was achieved either by microwaving/pressure cooking in 0.01 M sodium citrate buffer or by enzyme digestion with 0.02% pronase E (Sigma). Slides were thoroughly rinsed either under running tap water or in PBS to terminate retrieval of PBS. The assays used in these studies followed previously described protocols for immunostaining of TGFα, total EGFR, total erbB2, total erbB3 and phosphorylated ERK1/2 [37-39,41]. Cytoplasmic HRGβ1, phosphorylated membrane erbB2 and nuclear AKT immunostaining was carried out as detailed below.

Slides were incubated overnight in a sealed humidity chamber at room temperature either with rabbit anti-HRGβ1 antibody raised to a synthetic peptide from the COOH-terminal third of the epidermal growth factor homology region of human HRGβ1 protein (a kind gift from WJ Gullick, [42]) at 1/15 in 5% BSA/PBS, with rabbit anti-phospho-erbB2 antibody (06–229; Upstate Ltd, Chandlers Ford, Hampshire, UK) at 1/350 in PBS or with anti-phospho-AKT (New England Biolabs) at 1/50 in PBS. Following PBS rinsing and washing in DPC buffer (Euro/DPC Ltd., Llanberis, UK), all slides were covered for 2 hours with a 1/50 dilution of a goat anti-rabbit IgG peroxidase conjugate (A4914; Sigma). An omission of primary antibody was used as the control. Further confidence in the specificity of the assays was derived from control sections of each clinical sample that were incubated with a dilution of primary antibody matched to that of the paired tests but preabsorbed for 5 hours with appropriate blocking peptide (one part antibody:three parts peptide). The slides were then rinsed in PBS and DPC buffer. Signal detection was carried out for 10 minutes with freshly prepared diaminobenzidine tetrahydrochloride/hydrogen peroxide chromogen (Abbott Diagnostics ER-ICA kit, Maidenhead, UK). The resultant signal was enhanced for 7 minutes using 0.5%CuSO_4_·5H_2_O prepared in 0.85% NaCl and the slides were lightly counterstained using 0.5% methyl green. The slides were then dehydrated briefly before air-drying and clearing in xylene and a coverslip was positioned over the section using DPX mountant.

Cytoplasmic HRGβ1, membrane-phosphorylated erbB2 and nuclear-phosphorylated AKT immunostaining were assessed by two personnel using a dual-viewing attachment to an Olympus BH-2 light microscope (**Jencons Ltd, Leighton Buzzard, Bedfordshire, UK**). Estimates of the intensity of staining and the percentage of cells positively stained were determined using a minimum evaluation of 2,000 tumour cells per coverslip. These data were used to construct an H-score or a field staining index for each marker as described previously [43]

### Statistics

For immunocytochemical analysis of clinical samples, the Mann–Whitney U test was employed to compare median values between certain groups. The Spearman rank correlation test was also employed to determine the degree of association between the protein H-scores of all examined variables. For the Tam-R cell growth studies, overall differences between control and treatment groups were examined by one-way analysis of variance. Direct comparisons between control and treatment effects in Tam-R cells were determined using a Student *t *test with the Bonferroni adjustment factor. Differences were considered significant at the *P *< 0.05 level for all tests.

## Results

### HRGβ1, erbB3 and erbB4 expression

**Figure 1:**
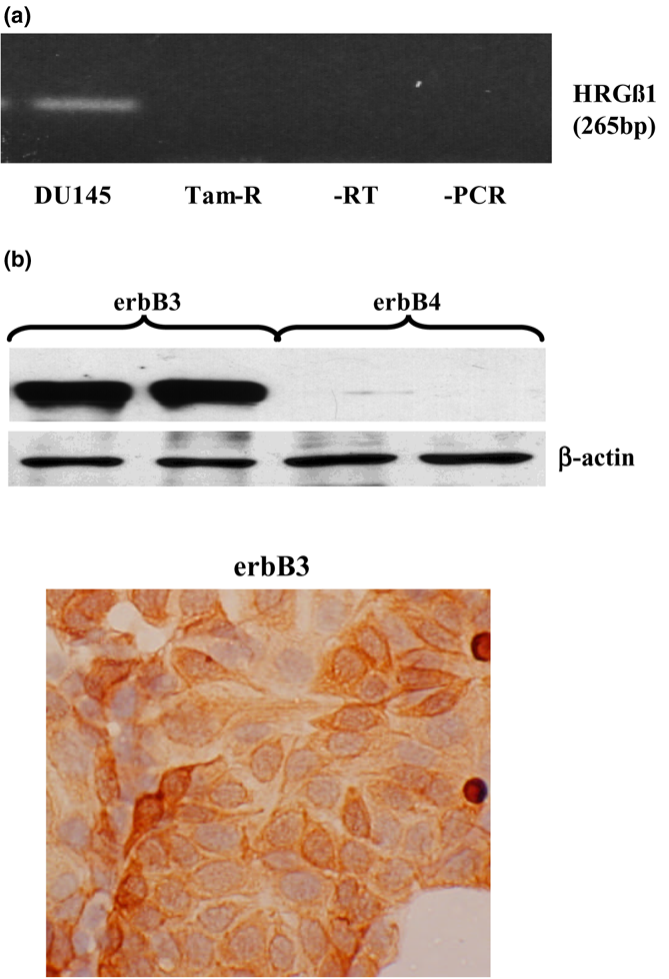
Expression of HRGβ1, erbB3 and erbB4 in tamoxifen-resistant MCF-7 cells. **(a) **RT-PCR analysis of basal HRGβ1 mRNA in DU145 prostate and tamoxifen-resistant MCF-7 (Tam-R) breast cancer cells. **(b) **Total erbB3 and erbB4 protein expression in Tam-R cells by western blotting and immunocytochemistry (original magnification, ×20). β-Actin protein expression was also assessed to demonstrate equivalent sample loading. Tamoxifen was present in Tam-R media. Data are representative of at least three separate experiments.

HRGβ1 mRNA could not be detected in Tam-R cells following 40 cycles of amplification; however, expression of this ligand was detected at the mRNA level in DU145 prostate cancer cells (positive control) (Figure 1a).

Western blotting and immunocytochemical studies demonstrated high expression levels of erbB3 protein in the Tam-R cell line, with high levels of membrane staining being observed in this cell line. Expression of erbB4, however, could barely be detected by western blotting (Figure 1b) and by immunocytochemistry (data not shown) in this cell line.

**Figure 2:**
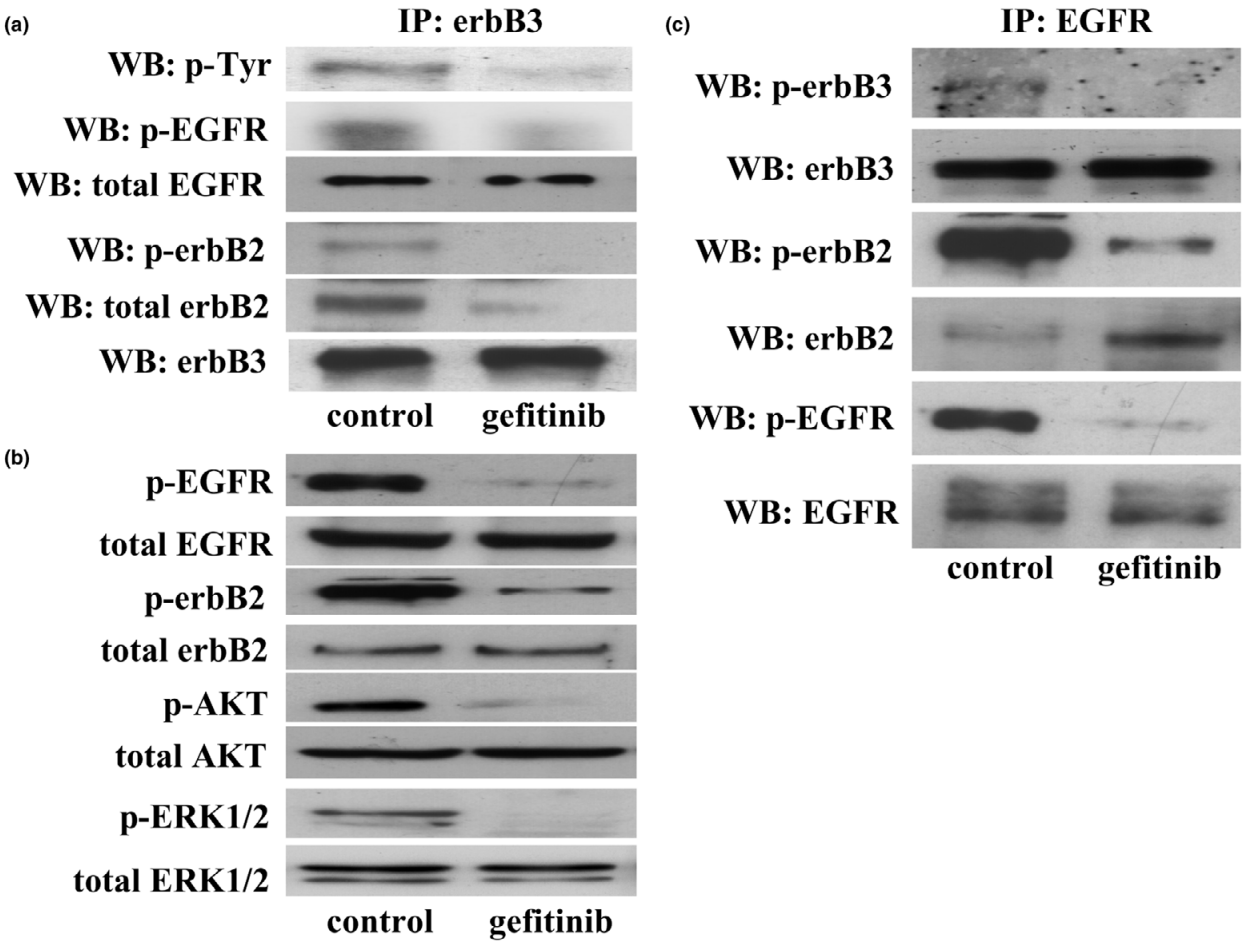
Effects of gefitinib on erbB receptor dimerization patterns and activity of associated downstream signalling elements. **(a) **Western blot (WB) analysis of phosphorylated epidermal growth factor receptor (p-EGFR), phosphorylated erbB2, phosphorylated tyrosine (Tyr) and total erbB3 protein expression following immunoprecipitation with total erbB3 antibody in tamoxifen-resistant MCF-7 (Tam-R) cells prior to and following incubation of Tam-R cells with gefitinib (1 μM) for 1 hour. **(b) **WB analysis of total and phosphorylated EGFR, erbB2, AKT and ERK1/2 protein expression in Tam-R cells prior to and following incubation with gefitinib (1 μM) for 1 hour. **(c) **WB analysis of total and phosphorylated EGFR, erbB2 and erbB3 protein expression following immunoprecipitation with total EGFR antibody in Tam-R cells prior to and following incubation with gefitinib (1 μM) for 1 hour. Tamoxifen (100 nM) was present in all studies. Data are representative of at least three separate experiments.

Under basal growth conditions, immunoprecipitation/western blotting studies revealed that phosphorylated levels of erbB3 were detectable in Tam-R cells (Figure 2a). Furthermore, the presence of low levels of phosphorylated erbB3/EGFR and erbB3/erbB2 heterodimers could be discerned (Figure 2a). Basal levels of phosphorylated AKT and ERK1/2 were also observed in the Tam-R cell line (Figure 2b). The high levels of basal phosphorylated erbB2 (Figure 2b) are primarily a consequence of EGFR/erbB2 heterodimerization and activation (Figure 2c) that we have previously shown to occur under basal growth conditions in this cell line [32].

### Effects of gefitinib

Gefitinib (1 μM) treatment promoted the formation of EGFR/erbB2 heterodimers, had no effect on levels of EGFR/erbB3 heterodimers and reduced erbB3/erbB2 heterodimers in the Tam-R cell line (Figure 2a,c). Gefitinib treatment also reduced basal phosphorylation levels of all heterodimers examined (Figure 2a,c). This inactivation of erbB heterodimers was associated with a reduction in basal EGFR, erbB2 and erbB3 phosphorylation levels in response to gefitinib in these cells (Figure 2a,b). Gefitinib was without effect on the total EGFR, erbB2 and erbB3 expression levels in this cell line (Figure 2a,b). Gefitinib treatment also potently inhibited basal ERK1/2 and AKT activity in both cell lines without exerting any effect on total protein expression levels (Figure 2b).

**Figure 3:**
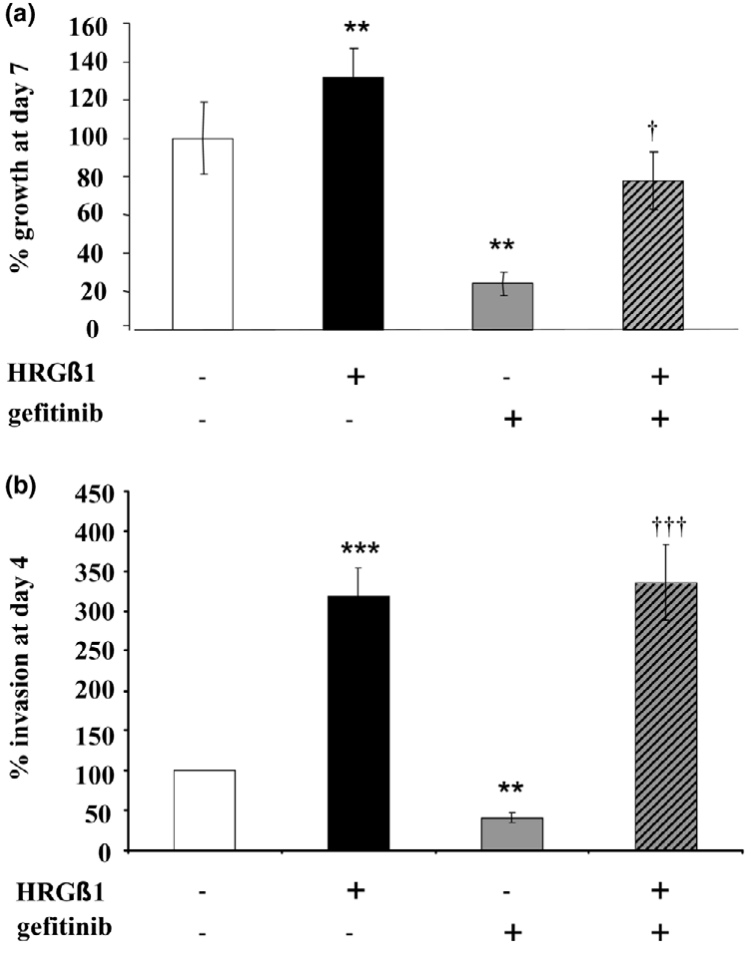
Effects of HRGβ1 and gefitinib on tamoxifen-resistant MCF-7 cell growth and invasion. **(a) **Growth and **(b) **invasive capacity of tamoxifen-resistant MCF-7 (Tam-R) cells, on day 7 for growth and on day 3 for invasion, following treatment with either HRGβ1 (10 ng/ml) or gefitinib (1 mM) or a combination of the two agents. Results expressed as the mean ± standard error of the mean of triplicate wells and are representative of at least three separate experiments. **P < 0.01 versus control, ***P < 0.001 versus control, ^†^*P *< 0.05 versus gefitinib, ^†††^*P *< 0.001 versus gefitinib.

As previously described, gefitinib was a potent inhibitor of both basal cell growth and invasion in Tam-R cells [32,35], reducing basal cell growth by approximately 75% at day 7 (*P *< 0.01, *n *= 4) and reducing basal cell invasion by approximately 60% at day 3 (*P *< 0.01, *n *= 3) (Figure 3).

### Effects of HRGβ1 in the absence and presence of gefitinib

**Figure 4:**
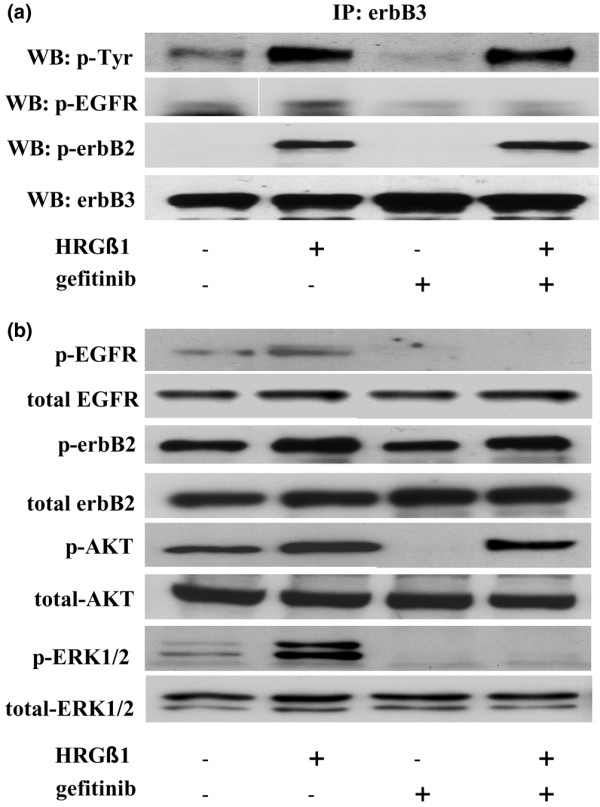
Effects of HRGβ1 and gefitinib on erbB receptor dimerization patterns and associated downstream signalling activity. **(a) **Western blot (WB) analysis of phosphorylated epidermal growth factor receptor (EGFR), phosphorylated erbB2, phosphorylated tyrosine (Tyr) and total erbB3 protein expression following immunoprecipitation with total erbB3 antibody in tamoxifen-resistant MCF-7 (Tam-R) cells prior to and following treatment with either gefitinib (1 μM) or vehicle control for 1 hour followed by HRGβ1 (10 ng/ml) for 5 minutes. **(b) **WB analysis of total and phosphorylated EGFR, erbB2, AKT and ERK1/2 protein expression in Tam-R cells prior to and following incubation with either gefitinib (1 μM) or vehicle control for 1 hour followed by either HRGβ1 (10 ng/ml) or vehicle control for 5 minutes. Tamoxifen was also present in all studies. Data are representative of at least three separate experiments.

Following treatment with HRGβ1 (10 ng/ml) the erbB3 receptor activity was dramatically increased in Tam-R cells, with HRGβ1 promoting activation of EGFR and erbB2 and promoting the formation of both active erbB3/erbB2 and erbB3/EGFR heterodimers in this cell line (Figure 4a,b). Under these same conditions basal expression levels of phosphorylated ERK1/2 and phosphorylated AKT were detectable in Tam-R cells as determined by western blotting, and the activity of both ERK1/2 and AKT were further increased following treatment of the cells with HRGβ1 (Figure 4b). These increases were not a consequence of increased total protein expression since both total ERK1/2 and AKT expression levels remained unchanged (Figure 4b).

In the presence of gefitinib, HRGβ1 was without effect on EGFR activity and did not promote the formation of activated erbB3/EGFR heterodimers in Tam-R cells. HRGβ1 was still capable, however, of activating erbB2 and of promoting formation of active erbB3/erbB2 heterodimers in the presence of the anti-EGFR agent in this cell line (Figure 4a,b). Furthermore, following gefitinib treatment, HRGβ1 was without effect on ERK1/2 activity whereas a HRGβ1-induced AKT activation was still apparent (Figure 4b).

Growth of Tam-R cells was significantly enhanced following HRGβ1 treatment, with an approximately 30% increase in proliferation being observed on day 7 of treatment compared with the control values (*P *< 0.01, *n *= 4; Figure 3). Similarly, Tam-R cell invasion was also significantly increased following HRGβ1 treatment, with approximately three times as many Tam-R cells invading through the artificial basement membrane following a 3-day treatment with this ligand (*P *< 0.001, *n *= 3; Figure 3). Gefitinib reduced Tam-R cell growth in response to HRGβ1, although a substantial and significant level of ligand-induced growth was still observed in the presence of this inhibitor (*P *< 0.05, *n *= 4; Figure 3). Gefitinib was without effect on HRGβ1-induced Tam-R cell invasion (Figure 3).

### Effect of trastuzumab alone and in combination with gefitinib

**Figure 5:**
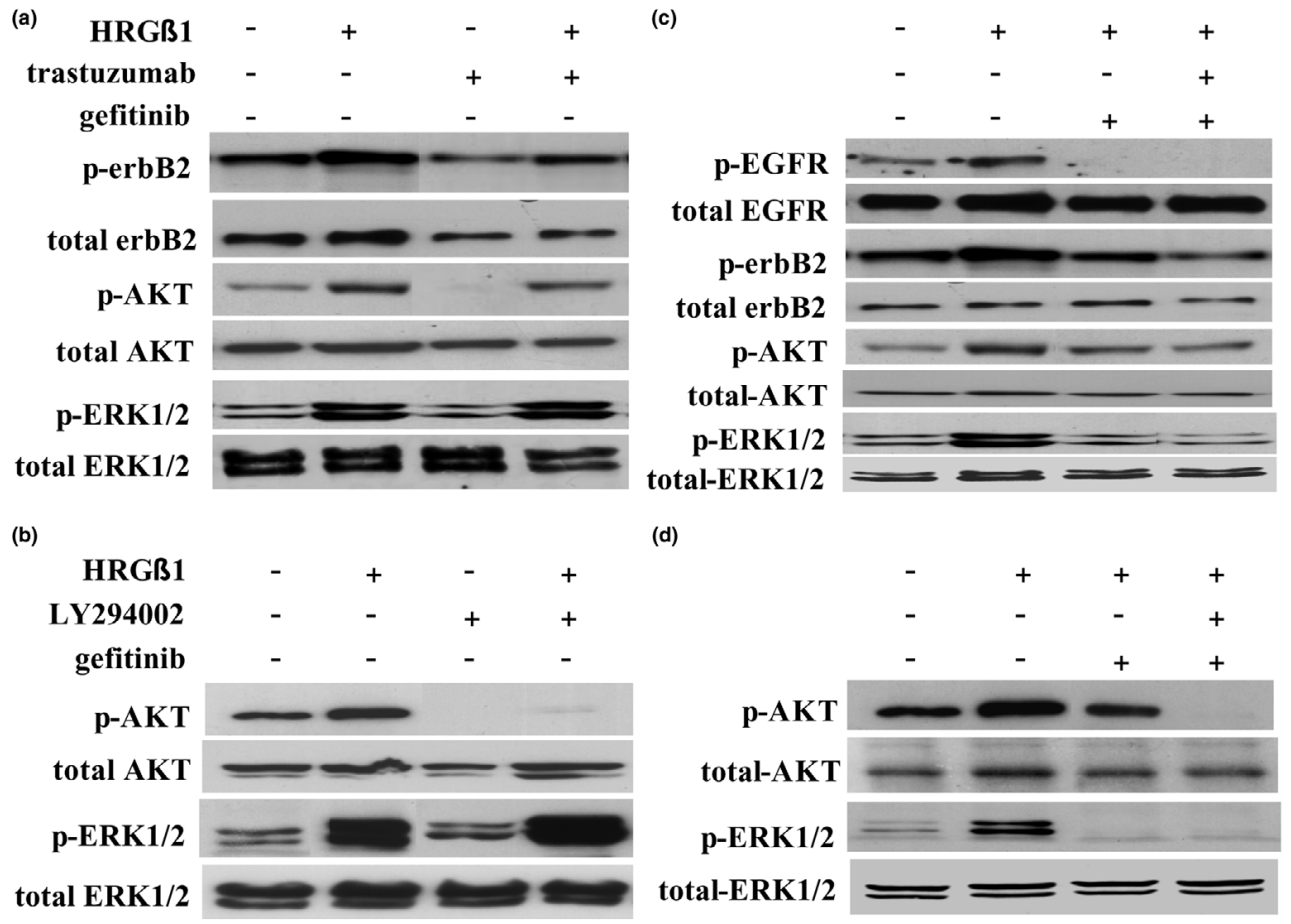
Effects of combining gefitinib with trastuzumab or LY294002 on HRGβ1-driven signalling in tamoxifen-resistant MCF-7 cells. Western analysis of total and phosphorylated epidermal growth factor receptor (EGFR), erbB2, AKT and ERK1/2 protein expression in tamoxifen-resistant MCF-7 (Tam-R) cells prior to and following incubation with either **(a) **trastuzumab (100 nM) or vehicle control for 7 days, **(b) **LY294002 (10 μM) or vehicle control for 1 hour, **(c) **gefitinib (1 μM), gefitinib in combination with trastuzumab or vehicle control for 7 days, and **(d) **gefitinib, gefitinib in combination with LY294002 or vehicle control for 1 hour, all followed by either HRGβ1 (10 ng/ml) or vehicle control for 5 minutes. Tamoxifen was also present in all studies. Data are representative of at least three separate experiments.

**Figure 6:**
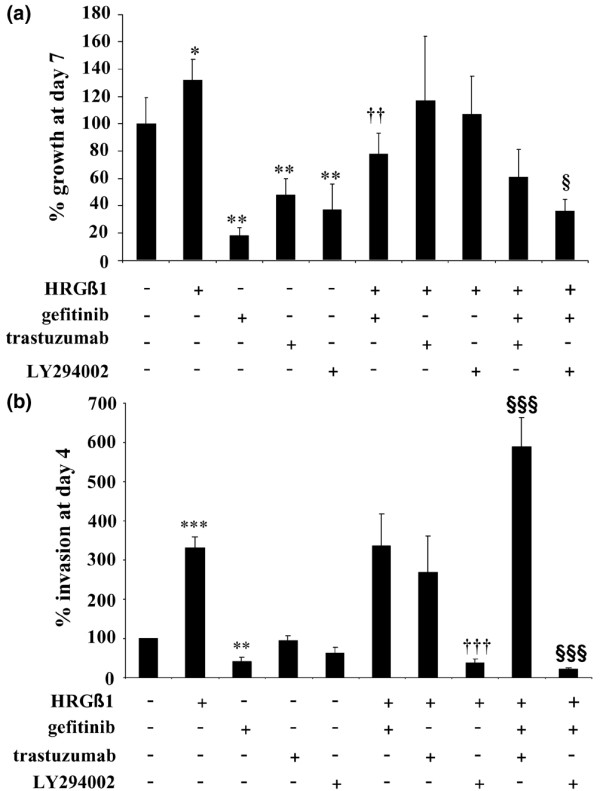
Effects of drug combinations on HRGβ1-driven tamoxifen-resistant MCF-7 cell growth and invasion. **(a) **Growth and **(b) **invasive capacity of tamoxifen-resistant MCF-7 (Tam-R) cells, on day 7 for growth and on day 3 for invasion, following treatment with either HRGβ1 (10 ng/ml), gefitinib (1 μM), trastuzumab (100 nM), LY294002 (10 μM) or a combination of these agents. Results expressed as the mean ± standard error of the mean of triplicate wells and are representative of three separate experiments. Tamoxifen was present in all studies. *P < 0.05, **P < 0.01 and ***P < 0.001 versus control; ^††^*P *< 0.01 and ^†††^*P *< 0.001 versus HRGβ1; ^§^*P *< 0.05 and ^§§§^*P *< 0.001 versus gefitinib + HRGβ1.

As previously reported [32], treatment of Tam-R cells with the anti-erbB2 monoclonal antibody trastuzumab (100 nM) reduced levels of basal phosphorylated and total erbB2, and this reduction was associated with a reduction in basal AKT and ERK1/2 activity and a significant inhibition of proliferative activity in these cells (*P *< 0.01, *n *= 6; Figures 5a and 6a). Trastuzumab was without effect, however, on the basal invasive capacity of Tam-R cells (Figure 6b).

The inhibitory effect of trastuzumab on cell signalling activity was overcome by HRGβ1, which promoted phosphorylation of erbB2, AKT and ERK1/2 in the presence of this antibody (Figure 5a). There was consequently no effect of trastuzumab on HRGβ1-induced Tam-R cell growth and invasion (Figure 6a,b).

In combination with gefitinib, trastuzumab treatment reduced HRGβ1-induced erbB2 and ERK1/2 activity but had only a minimal effect on AKT phosphorylation. This effect of trastuzumab and gefitinib in combination on the intracellular signalling activity was associated with a small, but not statistically significant, reduction in Tam-R cell growth (Figures 5c and 6a). There was a significant increase, however, in HRGβ1-mediated Tam-R cell invasion in response to this combination treatment (*P *< 0.001, *n *= 3; Figure 6b).

### Effect of LY294002 alone and in combination with gefitinib

The PI3K inhibitor LY294002 (10 μM) inhibited basal AKT activity and significantly reduced cell proliferation (*P *< 0.01, *n *= 6) in Tam-R cells (Figures 5b and 6a), but was without effect on basal ERK1/2 and invasion in this cell line (Figures 5b and 6b). HRGβ1 promoted ERK1/2 activity, but not AKT activity, in Tam-R cells in the presence of LY294002 (Figure 5b), and this promotion was associated with enhanced Tam-R cell growth equivalent to that seen in response to HRGβ1 alone (Figure 6a). LY294002, however, prevented HRGβ1-induced Tam-R cell invasion (*P *< 0.001, *n *= 3; Figure 6b).

LY294002 in combination with gefitinib reduced HRGβ1-induced activation of both ERK1/2 and AKT (Figure 5d), and significantly reduced HRGβ1-induced cell growth (*P *< 0.05, *n *= 6; Figure 6a). Furthermore, the combination of gefitinib and LY294002 virtually abolished HRGβ1-driven Tam-R cell invasion (*P *< 0.001, *n *= 3; Figure 6b).

### Clinical associations

**Figure 7:**
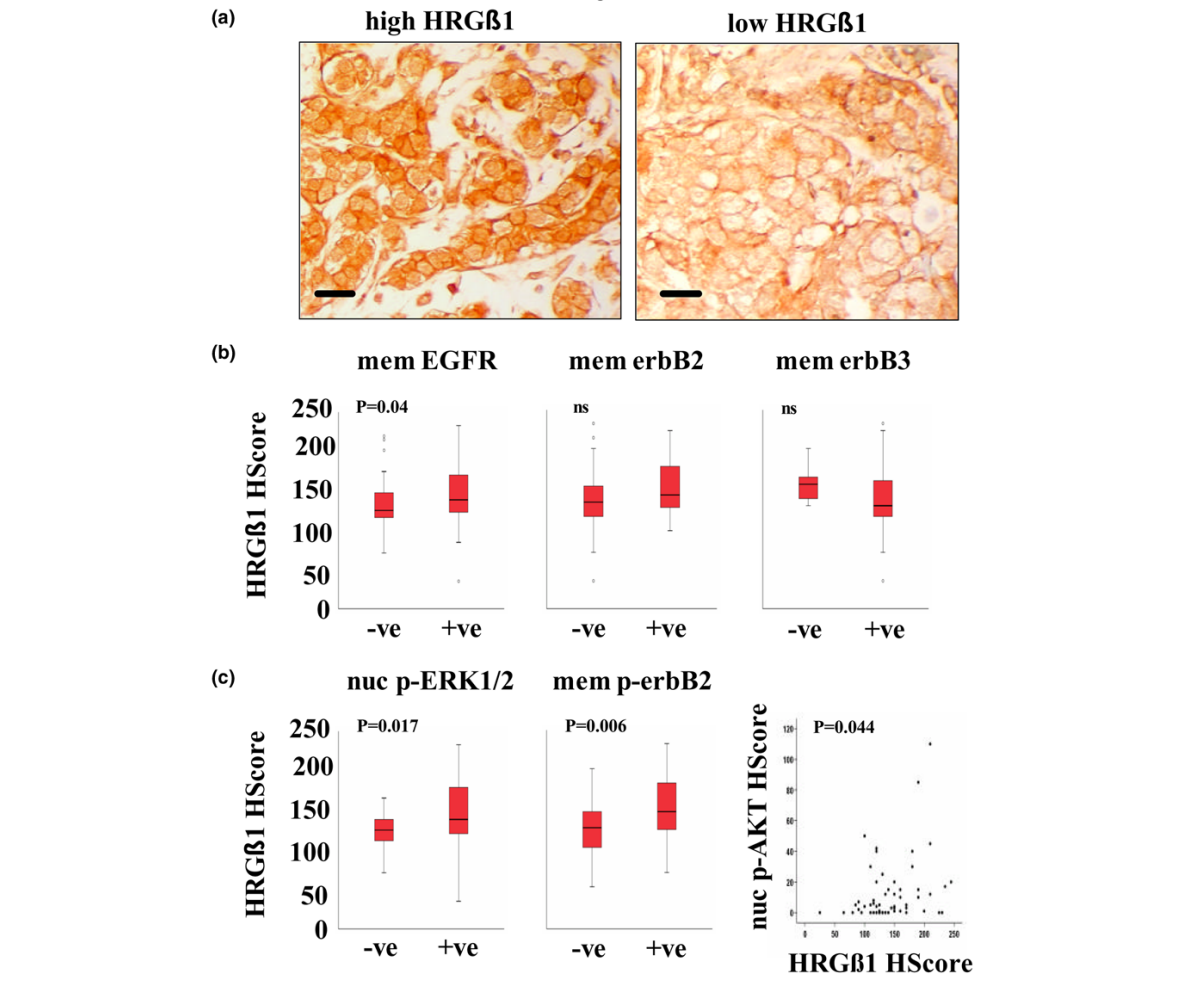
Immunohistochemical staining for HRGβ1 in primary breast cancer specimens. **(a) **Examples of high and low HRGβ1 expression. Scale bars = 20 μm. **(b) **Box-plots illustrating cytoplasmic HRGβ1 immunohistochemistry assessed by H-score analysis in membrane (mem) epidermal growth factor receptor (EGFR)-negative, erbB2-negative and erbB3-negative primary breast cancer versus membrane EGFR-positive, erbB2-positive and erbB3-positive primary breast cancer. A significant positive correlation between cytoplasmic HRGβ1 expression and membrane erbB receptor positivity was only seen with EGFR (Mann–Whitney U test, *P *= 0.04). **(c) **Box-plots illustrating cytoplasmic HRGβ1 immunohistochemistry assessed by H-score analysis in membrane (mem) phosphorylated erbB2-negative versus membrane phosphorylated erbB2-positive primary breast cancer and in nuclear (nuc) phosphorylated ERK1/2-negative versus nuclear phosphorylated ERK1/2-positive primary breast cancer (Mann–Whitney U test, *P *= 0.006 and *P *= 0.017, respectively), and scatter plot illustrating the significant positive correlation between expression levels of nuclear phosphorylated AKT and cytoplasmic HRGβ1 in the same samples (Spearman rank test, *P *= 0.044).

All clinical breast samples examined exhibited tumour epithelial staining for HRGβ1. This staining ranged from barely detectable to highly positive and was predominantly cytoplasmic, although a low level of membrane staining was observed (Figure 7a). There was no evidence of nuclear staining. Staining within individual tumours was reasonably homogeneous and control sections remained unstained.

Statistical analyses using either the Mann–Whitney U test and/or the Spearman rank correlation test were applied to all patient samples to determine the relationships between HRGβ1 immunostaining and the tumour grade, the survival time and the oestrogen receptor, EGFR, erbB2, MAPK, AKT, Ki-67, menopausal and metastatic status. Appropriate cutoff or marker status values for cytoplasmic TGFα, nuclear oestrogen receptor, MAPK and total membrane EGFR, erbB2 and erbB3 were as described previously [38-40]. Status values for phosphorylated membrane erbB2 were based on true cutoff values where the cutoff value was zero (no detectable immunostaining), whereas for phosphorylated nuclear AKT the median value was chosen.

Examination of the degree of HRGβ1 immunostaining in all patients identified no statistically significant correlations between HRGβ1 expression and the tumour grade, menopausal, nuclear oestrogen receptor, cytoplasmic TGFα, membrane erbB2 and membrane erbB3 status values. Statistically significant and positive correlations, however, were observed between total membrane EGFR, phosphorylated membrane erbB2 and phosphorylated nuclear MAPK status values and HRGβ1 positivity (Mann–Whitney U test, *P *= 0.04, *P *= 0.006, *P *= 0.017, respectively; Figure 7b,c). Moreover, application of the Spearman rank correlation test to these data also indicated a direct correlation between the levels of cytoplasmic HRGβ1 and phosphorylated nuclear AKT immunostaining (*P *= 0.044), as illustrated in Figure 7c. Furthermore, HRGβ1 levels were shown to be significantly higher in tumours from patients with distant metastatic deposits compared with tumours from patients with only locally advanced disease (Mann–Whitney U test, *P *= 0.013). Using the median as a cutoff value for HRGβ1 expression, there was also a trend towards a poorer prognosis for patients expressing higher levels of HRGβ1, although this trend did not reach statistical significance.

## Discussion

Despite the clear therapeutic promise of anti-EGFR therapies in the preclinical setting, with agents such as gefitinib potently inhibiting growth of a range of human cancer cell lines that express a functional EGFR, there is increasing evidence – from both preclinical and clinical studies – of primary/*de novo *and acquired resistance to these inhibitors [11-13]. A key mechanism implicated in resistance to anti-EGFR agents is activation of the PI3K/AKT signalling pathway either as a result of loss of phosphatase and tensin homologue function or of enhanced insulin-like growth factor type 1 receptor activity [12,15-19]. More recently it has been reported that, in human MKN7 gastric cancer cells, HRGβ1 can also circumvent the antiproliferative action of the selective EGFR-TKI CGP59326 through promotion of erbB3/erbB2 heterodimerization and activation of the PI3K signalling pathway [44]. In the present study we have, for the first time, examined whether HRGs can similarly promote erbB3/PI3K/AKT signalling and effectively circumvent the inhibitory effects of the anti-EGFR agent gefitinib on the growth and invasion of an EGFR-positive Tam-R breast cancer cell line [32].

In agreement with our previous findings, the Tam-R cell line was found to express erbB3 at high levels, whereas only minimal expression of erbB4 was observed in this cell line [32]. erbB3 expression was localized primarily at the cell membrane, although some cytoplasmic staining for this receptor was also observed by immunocytochemistry. We also attempted to assess expression of HRGβ1, a key erbB3 ligand, in this cell line by RT-PCR but were unable to detect a signal. It has previously been reported that MCF-7 cells do not synthesize HRGs [45], and our findings now indicate that the inability to generate this ligand is maintained through acquisition of tamoxifen resistance in this cell line. The lack of any autocrine release and action of heregulins by the Tam-R cell line would explain the low levels of erbB3/erbB2 heterodimers detected under basal growth conditions. Although low levels of erbB3 activity were also observed under these conditions, this is a result of the formation of erbB3/EGFR heterodimers – most probably a consequence of the autocrine release and action of the EGFR ligand amphiregulin, which we have previously shown to be synthesized and released by this cell line [34]. Indeed, this hypothesis is supported by the fact that basal erbB3 activity could be reduced by treatment of the cells with the selective EGFR tyrosine kinase inhibitor gefitinib. Basal erbB4 activity and heterodimerization could not be detected in these cells (data not shown).

In agreement with our previous findings, treatment of Tam-R cells with gefitinib (1 μM) blocked the basal phosphorylation of EGFR, reduced the basal formation of active EGFR/erbB3 heterodimers and inhibited the basal activation of both AKT and ERK1/2 [32,34,41]. The reduced basal EGFR/MAPK/AKT signalling activity in response to gefitinib was also associated with a significant reduction in both Tam-R cell proliferation and invasion, again as previously reported, confirming the central role played by EGFR signalling in this cell line [32,35]. Interestingly, we also found that gefitinib was capable of reducing phosphorylation levels of erbB2, an observation we have reported on previously [32]. Gefitinib is highly selective for EGFR (median inhibitory concentration = 0.033 μM) but can inhibit erbB2 tyrosine kinase activity with a median inhibitory concentration of approximately 3–4 μM [46]. At a concentration of 1 μM, however, gefitinib should have little direct effect on erbB2 tyrosine kinase activity in this study. More recently it has been proposed that gefitinib can indirectly inhibit erbB2 activity through inducing the formation of inactive EGFR/erbB2 heterodimers in erbB2-overexpressing BT-474 cells [47]. In agreement with Anido and colleagues [47], we found that gefitinib promoted the formation of inactive EGFR/erbB3 and EGFR/erbB2 heterodimers in our Tam-R cells. This ability of gefitinib to sequestrate erbB2 into an inactive complex with EGFR thus prevented activation of erbB2 through dimerization with other erbB family members.

A possible consequence of the sequestration of erbB2 and erbB3 into inactive heterodimers with EGFR is a reduction in the availability of erbB2 and erbB3 within the cell. Indeed, it has been reported that basal levels of erbB2/erbB3 heterodimers are greatly reduced following treatment of BT-474 breast cancer cells with gefitinib, resulting in a substantial blunting of response to HRGβ1 treatment [47]. In agreement with these findings we demonstrated in the present study that levels of erbB2/erbB3 heterodimers were reduced following gefitinib treatment in Tam-R cells and that gefitinib prevented HRGβ1-induced activation of EGFR and ERK1/2 while reducing HRGβ1-induced activation of erbB2 and AKT in this cell line. Since this effect on erbB2 and AKT activity cannot be attributed to the tyrosine kinase inhibitory activity of gefitinib because erbB3 is kinase dead [48] and, as mentioned previously, far higher concentrations of this inhibitor are required to block erbB2 activity, it is more probably a consequence of the reduced erbB2 and erbB3 receptor availability due to gefitinib-induced sequestration with EGFR. It should also, however, be noted that although HRGβ1 signalling is reduced by gefitinib treatment in Tam-R cells, this ligand was still capable of promoting the formation of active erbB2/erbB3, activating AKT signalling and potently driving cell growth.

These findings clearly indicate that although EGFR is the dominant pathway driving growth in Tam-R cells, if this pathway is blocked then these cells are capable of utilizing alternative signalling pathways such as the HRGβ1/erbB3/AKT pathway to circumvent this blockade and to very effectively maintain cell growth. Such a resistance mechanism to anti-EGFR therapy is not unique to Tam-R cells as it has also been shown in MKN7 gastric cancer cells that HRGs can overcome the growth inhibitory actions of the EGFR tyrosine kinase inhibitor CGP59326 through activation of downstream AKT and ERK1/2 signalling pathways [44]. It is also important to point out that although HRGβ1 only partially recovered Tam-R cell growth in the presence of gefitinib, this ligand fully circumvented the blockade of Tam-R cell invasion generated by this anti-EGFR agent. HRGβ1 signalling therefore appears as effective as EGFR signalling in driving invasion of Tam-R cells, suggesting that targeting EGFR in the presence of HRGs will have minimal effect on the aggressive phenotype of this cell line.

We next examined the effect of targeting components of the HRGβ1 signalling pathway in Tam-R cells to assess whether such a strategy, in combination with gefitinib, would more effectively reduce growth and invasive capacity. We initially targeted erbB2 using the monoclonal antibody trastuzumab. It has previously been demonstrated that combined targeting of EGFR and erbB2 is more effective than targeting either agent alone in inhibiting growth of erbB2-overexpressing breast cancer cells [44,49-51]. We similarly found, in the present study, that Tam-R cells demonstrated a small reduction in HRGβ1-induced erbB2 and ERK1/2 signalling following treatment with trastuzumab and gefitinib in combination compared with gefitinib alone. This effect of the combination treatment on HRGβ1 signalling activity was also associated with a small but not significant reduction in HRGβ1-driven Tam-R cell growth when compared with the effects of gefitinib alone. This combination strategy, however, was without further significant effect on HRGβ1-induced AKT activity compared with the effects of gefitinib alone, and induced a substantial and significant enhancement of, rather than an inhibition of, HRGβ1-driven Tam-R cell invasion. Targeting erbB2 with trastuzumab in combination with gefitinib to block HRGβ1 signalling therefore had only a minimal effect on cell growth and also had the potential to make the cells substantially more aggressive in nature.

Interestingly, a recent phase I–II clinical study assessing the effect of trastuzumab in combination with gefitinib in patients with erbB2-overexpressing metastatic breast cancer reported that the time to progression was shorter for the combination compared with trastuzumab alone [52]. Furthermore, this combination treatment was shown to increase erbB3 activity in erbB2-overexpressing breast cancer cells [52]. Taken in conjunction with the present findings it is possible that HRGβ1/erbB3-mediated signalling in the presence of trastuzumab and gefitinib may maintain cell growth and promote enhanced invasive features within the tumour cells, potentially explaining the reduced time to progression observed in patients receiving this treatment regime.

A more effective combination therapy that inhibited both HRGβ1-driven Tam-R cell growth and invasion was gefitinib in conjunction with the PI3K inhibitor LY294002. Treatment of Tam-R cells with this combination potently knocked out activation of both AKT and ERK1/2. These findings, alongside the inability of trastuzumab and gefitinib to block HRGβ1-induced AKT activity, would suggest that the PI3K/AKT signalling pathway is an important mediator of HRGβ1-driven Tam-R cell invasion and appears to be activated by an EGFR-independent and erbB2-independent mechanism in these cells. Indeed, heregulin-induced PI3K/AKT signalling has been implicated as a dominant signalling pathway driving cell invasion in a range of breast cancer cells [25,26,28,31]. It should also, however, be noted that the enhanced invasive capacity of the Tam-R cell line in response to trastuzumab and gefitinib in combination was not associated with an increase in AKT activity; indeed, if anything, AKT activity was slightly reduced in the presence of the two inhibitors. As we have found that this enhanced invasive response to HRGβ1 in the presence of both gefitinib and trastuzumab remains sensitive to inhibition by LY294002 (data not shown), it is therefore probable that other PI3K-dependent mechanisms are also involved in mediating this process. Identifying the exact mechanisms involved in the EGFR/erbB2-independent HRGβ1-mediated activation of PI3K-dependent signalling and in the enhanced invasive behaviour in our Tam-R cell line remains a subject of intense investigation within our laboratory.

Finally, we examined whether the HRGβ1-mediated gefitinib-resistant mechanism identified in our Tam-R cells may also be functional within breast tumours by assessing HRGβ1 expression in a set of 77 breast cancer tissue samples from breast cancer patients who had received no previous therapies. We found that all samples expressed predominantly cytoplasmic HRGβ1, with this staining being highly heterogeneous between tumour samples. These findings are in agreement with other reports assessing HRGβ1 expression in primary breast cancer specimens [23,24], with both of these studies demonstrating exclusively cytoplasmic staining for this ligand. Interestingly, in the present study the highest expression levels of cytoplasmic HRGβ1 were found in patients with tumours that expressed high levels of membrane EGFR and increased activity of the EGFR-associated signalling elements, membrane erbB2, nuclear MAPK and nuclear AKT.

Interestingly, there was no correlation between expression of cytoplasmic HRGβ1 expression and either membrane erbB2 or erbB3 in these samples. It should be noted that Visscher and colleagues [23] were unable to identify any correlations between EGFR and HRG expression in their primary tumour specimens; however, this may simply reflect the very small dataset (34 samples) used in the study [23]. A profile of high EGFR expression and increased activation of downstream MAPK and AKT signalling pathways, which mimics the expression profile observed in our Tam-R model, would suggest that these patients may benefit from anti-EGFR-targeted therapy. Since HRGβ1 is also highly expressed in these samples, however, our model would predict that targeting the EGFR with agents such as gefitinib would not prove effective due to the ability of HRGβ1 to overcome EGFR blockade and to potently drive resistant growth and invasion. Indeed, HRGβ1 levels were also shown to be significantly higher in distant metastatic versus locally advanced patients, suggesting a possible link between this ligand and the invasive capacity of tumour cells.

## Conclusion

We have demonstrated that HRGβ1 can partially overcome the inhibitory effects of gefitinib monotherapy on growth and invasion of tamoxifen-resistant MCF-7 breast cancer cells through promotion of erbB3/erbB2 heterodimerization and activation of the PI3K/AKT signalling pathway. Furthermore, such a mechanism may be functional in the clinical setting as HRGβ1 is highly expressed in some EGFR-positive breast cancers, thus providing a possible *de novo *resistance mechanism to any utilized anti-EGFR therapy. Combination therapy targeting HRGβ1 signalling alongside EGFR blockade can enhance the effectiveness of agents such as gefitinib on growth and invasion; however, the findings with trastuzumab and gefitinib in combination, where the minimal benefit of growth inhibition is offset by enhanced invasive capacity, suggest that rather than simply focusing on cell growth we should also consider other properties of the cancer phenotype if we are to fully understand the impact of these therapies on the cancer cell.

## Abbreviations

BSA = bovine serum albumin; EGFR = epidermal growth factor receptor; FCS = foetal calf serum; HRG = heregulin; MAPK = mitogen-activated protein kinase; PBS = phosphate-buffered saline; PCR = polymerase chain reaction; PI3K = phosphatidylinositol 3-kinase; Tam-R = tamoxifen-resistant MCF-7; TBS = Tris-buffered saline; TGFα = transforming growth factor alpha.

## Competing interests

SEH, JMWG and RIN are in receipt of funding from AstraZeneca, and RIN is also a member of an advisory board for AstraZeneca. The remaining authors declare that they have no competing interests.

## Authors' contributions

IRH conceived the study and participated in its design and execution, and in drafting the manuscript. JMK carried out the PCR, participated in the immunoprecipitation and western blotting studies, and helped to draft the manuscript. SEH carried out the invasion assays. DB performed all cell cultures and carried out the growth studies. JMWG carried out the immunocytochemistry and helped to draft the manuscript. JFR and IOE provided the clinical breast cancer samples. RIN participated in the design and coordination of the study and helped to draft the manuscript. All authors read and approved the manuscript.
