# Correction to: Heregulin β1 drives gefitinib-resistant growth and invasion in tamoxifen-resistant MCF-7 breast cancer cells

**DOI:** 10.1186/s13058-018-0999-6

**Published:** 2018-08-30

**Authors:** Iain R. Hutcheson, Janice M. Knowlden, Steve E. Hiscox, Denise Barrow, Julia M. W. Gee, John F. Robertson, Ian O. Ellis, Robert I. Nicholson

**Affiliations:** 1 Tenovus Centre for Cancer Research, Welsh School of Pharmacy, Cardiff University, Redwood Building, King Edward VII Avenue, Cardiff, CF10 3XF UK; 2 Professorial Unit of Surgery, Nottingham City Hospital, Hucknall Road, Nottingham, NG5 1PB UK

## Correction

After the publication of this work [1], an error was noticed in Fig. 2b and Fig. 4b as well as Fig. 5d. and Fig. 5d. Images of the ERK1/2 blots were accidentally duplicated. In Fig. 5a. and Fig. 5c., the last lane for p-ERK1/2 was mistakenly cropped out of the final image. The original blot for Fig. 4b., “total EGFR” (or lane 2) is shown below to avoid any misunderstanding of the data. We apologize for this error, which did not affect any of the interpretations or conclusions of the article.

Fig. 4b. Uncropped original blot of “total EGFR” suggests that total EGFR levels increase with gefitinib treatment. However, increases in EGFR expression in response to gefitinib, are not seen across the study, which would suggest this increase is simply an anomaly.
